# Current Concepts: Corrective Osteotomy for Extra-Articular Deformity Following a Distal Radius Fracture

**DOI:** 10.7759/cureus.47019

**Published:** 2023-10-14

**Authors:** Samuel C Haines, Alasdair Bott

**Affiliations:** 1 Trauma and Orthopaedics, Bristol Royal Infirmary, Bristol, GBR; 2 Trauma and Orthopaedics, Southmead Hospital, Bristol, GBR

**Keywords:** druj injury, wrist fractures, extra-articular, corrective osteotomy, distal radius fractures

## Abstract

Fracture malunion alters wrist and distal radioulnar joint (DRUJ) biomechanics, resulting in incongruence and instability of the DRUJ. Selected patients with painful functional limitation and significant deformity of the radius, but without advanced degenerative joint disease, may benefit from corrective distal radial osteotomy. Non-union and complications arising from metalwork are the most common reasons for reoperation. Surgeons should have a good understanding of risks and complications in order to fully inform their patients and manage expectations. This article reviews the biomechanical effects of radial malunion and the current concepts for treatment. Distal radial osteotomy is suitable for symptomatic patients with angular radial deformity and shortening. Evidence supports a volar approach without bone grafts for modest corrections. Bone grafts or synthetic bone substitutes are appropriate for larger corrections. Functional improvements are reported regardless of technique. Despite a high complication rate, patient satisfaction with the corrective radial osteotomy is high.

## Introduction and background

Distal radial fractures are the most common of all adult orthopaedic injuries, accounting for between 18% and 25% of all fractures [1]. The United Kingdom Distal Radius Acute Fracture Fixation Trial (DRAFFT) and British Orthopaedic Association Standards for Trauma guidance present strong evidence in support of a move back towards more conservative treatment of extra-articular fractures, particularly in those over 65 years of age with minimal deformity [2-3].

Although significant deformity is recognised as a relative indication for surgery, there is insufficient evidence to demonstrate a clear association between any measured radiological parameters and patient-rated outcomes [3]. The correlation between malunion and the development of symptomatic osteoarthritis remains unclear [4].

Despite the majority of patients regaining satisfactory wrist function, 23%-31% of distal radial fracture malunions cause persistent limitation of function due to symptoms of pain, loss of range of motion, and grip strength [5-7].

This article reviews the current understanding of the biomechanical effects of radial malunion, indications for corrective distal radial osteotomy, and options for operative technique.

## Review

Biomechanics of bony deformity

**Figure 1:**
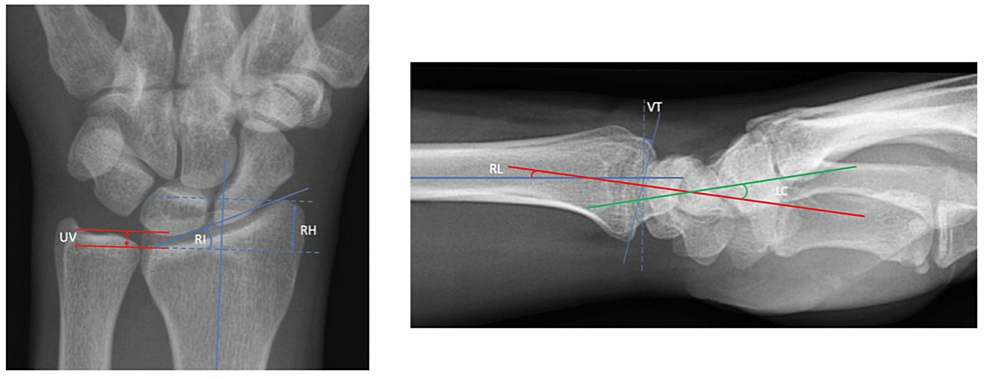
Established radiographic parameters Left: Posteroanterior radiograph ulnar variance (UV) = -2mm to +2mm; radial inclination (RI) = 22 degrees; radial height (RH) = 12mm; right: Volar tilt (VT) = 11 degrees; radiolunate angle (RL) = 10 +/- 6 degrees; lunocapitate angle (LC) = 0 +/- 12 degrees

Normal wrist anatomy, biomechanics, and established radiographic parameters (Figure 1) have been extensively researched and reported [8-11]

Malunion of the distal radius can result in multiplanar deformities and adaptive carpal malalignment (carpal instability adaptive (CIA)). Dorsal angulation greater than 25-degrees leads to malalignment at the radiocarpal joint; less severe radial deformities are associated with midcarpal malalignment and a dorsal intercalated segmental instability (DISI) deformity [12-13].

Deformities with relative radial shortening and dorsal tilt are believed to be of most importance due to the effect they have on the distal radioulnar joint (DRUJ). Distal radioulnar joint disruption remains the main source of pain and stiffness in pronosupination [14-16]. Radial shortening creates increased contact pressure along the ulnar-sided sling of the triangular fibrocartilage complex (TFCC). The degeneration of TFCC secondary to symptomatic ulnocarpal impaction is also associated with erosion of the lunate and lunotriquetral ligaments [9].

**Figure 2:**
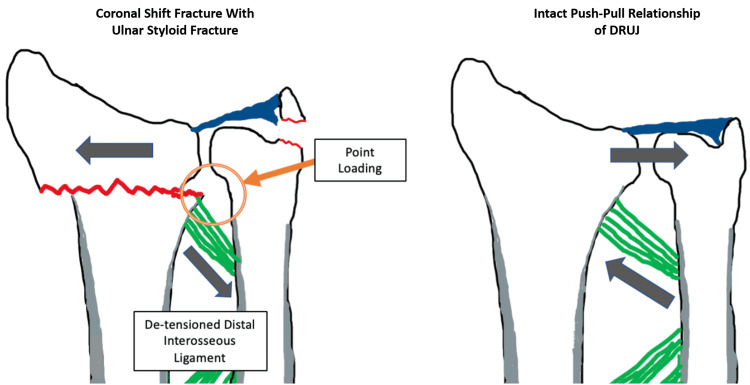
DRUJ schematics Left: coronal shift fracture; right: normal push-pull relationship of DRUJ DRUJ: distal radioulnar joint

Rotational deformities are often underappreciated, but they were reported in 23 of 37 malunions when measured using computed tomography (CT) [17]. Coronal shifts with associated ulnar styloid fractures can result in de-tensioning of the TFCC through loss of the push at DRUJ and pull of the ulnar styloid [15] and can lead to point loading of the proximal radius against the ulna (Figure 2).

Loss of radial inclination probably causes less severe functional impairment [9].

The malunited distal radius alters the congruity of the DRUJ articular surface, and in vivo studies demonstrate a reduction in the contact area of the DRUJ when compared to their cohort’s uninjured wrists, from 215 mm^2^ to 155 mm^2^, and a proximal shift of maximum articular contact pressure [18].

Biomechanics of TFCC and DRUJ disruption

It should be remembered that ulnar styloid fractures accompany 51% to 65% of distal radius fractures. These fractures represent part of a structural injury to the TFCC and stabilisers of the DRUJ. Further, acute tears of the TFCC were present in 11%-19% of cases at the time of arthroscopically-assisted fracture fixation in patients without ulnar styloid fractures [10, 19-20]. Traumatic TFCC injuries compound radial shortening, with reports of isolated TFCC injuries causing 0.5 to 3.0 mm of proximal radial migration [10]. Crisco et al. studied malunion kinematics and the distortion of volar and dorsal radioulnar ligaments in malunited fractures using three-dimensional (3D) CT scans and computer modelling [21]. They found dorsal angulation and shortening caused stretching of the dorsal ligamentous structures, which was worst in pronation. These altered strains on the tissues of the TFCC can present as DRUJ instability. Treatment of altered soft tissue structures may therefore be as important as correction of abnormal bone anatomy [18,21].

Patient evaluation

A detailed patient history and examination are critical to determining the cause of symptoms and appropriate management. Patient history should include the nature of the patient’s employment and functional demands, a detailed pain history, and details of early fracture management [22-23].

A history of comorbid medical conditions, such as osteoporosis, diabetes, and long-term use of corticosteroids or other immune modulators, should be sought as they may adversely affect bone quality, soft tissue healing, and bone union [22]. In particular, the smoking status of the patient should be ascertained, given its impact on microcirculation and union rates [23-24].

A clinical examination is vital in determining the source of pain. A full neurological exam should exclude the presence of carpal tunnel syndrome, present in up to 17% of patients, and features of complex regional pain syndrome [25].

The range of wrist movement should be recorded in the following three planes of movement: (1) Flexion/extension: Wrist flexion may be limited by malunions with dorsal extension. (2) Radial/ulnar deviation: Radial shortening and loss of radial inclination can precipitate ulnocarpal impaction. Patients experience ulnar-sided pain made worse by an ulnar deviation of the wrist. (3) Rotation: The limitation of pronosupination is usually due to soft tissue distortion rather than bony impingement, which may be judged by the presence of a ‘hard end-point’ block to rotation [26].

A clinical examination should include an assessment of DRUJ stability. The presence of clicking or crepitus may indicate TFCC pathology or osteoarthritis. Many authors report poor grip strength in affected patients; thus, objective measurement using a dynamometer can prove a useful way of assessing response to treatment [6,27-28].

Investigation

A review of existing imaging may aid in understanding the injury pattern and chronology. A plain X-ray is the first line of investigation. Orthogonal views at the wrist and posteroanterior radiographs should be taken with the arm abducted to 90 degrees and the elbow flexed to 90 degrees in order to make reproducible judgements about ulnar variance [28], as this can differ greatly among individuals and hence should be evaluated by comparison to the contralateral, uninjured extremity, providing a template for reconstruction [29-30].

Widening of the articular surface of the distal radius can indicate intra-articular extension of the original injury and therefore should alert the clinician to the possibility of degenerative joint disease [4,31-32].

In most cases, the nature of the deformity can be accurately judged using plain film radiography, although rotational deformities are difficult to appreciate [33]. For this reason, a CT scan can help in the assessment of rotation, congruity of DRUJ, and pre-operative planning. In cases of extreme deformities, operative planning can be aided using 3D printing based on CT reconstruction. This can be used as a practice template [34].

An MRI can aid in the diagnosis of associated pathology in the wrist, particularly ulnocarpal abutment and localised tenosynovitis. Magnetic resonance imaging arthrography has been reported to have a sensitivity and specificity of 90%-92% and 89%-70%, respectively, in the diagnosis of TFCC tears [35-36]. Rupture of the deep fibres of the TFCC is best seen on MRI unless the DRUJ is investigated arthroscopically. Arthroscopic evaluation of cartilage integrity may be used in selected cases to guide decision-making between corrective osteotomy or salvage procedures [37].

Non-surgical management

For functional limitations, a programme of focused physiotherapy to stretch and strengthen the wrist should be first-line therapy. The majority of malunions are well tolerated, especially in lower-functional-demand individuals. Activity modification, in conjunction with the use of splints and supports, may avert the need for surgical intervention. Targeted steroid injections may provide some degree of symptom control for non-surgical candidates.

Surgical indications

Surgical candidates are those with persistent pain and disability consistent with their deformity, despite a trial of non-operative treatment. Careful consideration should be given to the timing of surgery. A subacute correction may be considered before fracture union occurs. For established fracture malunion, most authors agree that by six months post-injury, patients who remain symptomatic should be considered. Mahmoud et al. reported improved post-operative Disabilities of Arm, Shoulder, and Hand (DASH) scores with a shorter interval between injury and correction [38].

The radiological criteria for malunion are variable across the literature; despite this, intervention may be considered in cases of radial height < 7 mm, positive ulna variance of >3- 5 mm, and radial tilt > 15° dorsal (or >20° volar tilt) [11,39-40]. Assessments should be made with reference to the contralateral, uninjured limb. Osteotomy of the distal radius is suitable when there is an angular deformity that would not be treated more reliably with ulnar shortening osteotomy alone.

Even with careful pre-operative planning, corrective surgery is challenging and does not restore a ‘normal’ wrist [5,41]. Patients considered for intervention should be fully informed regarding recovery timescales, likely outcomes, and potential complications. Patients without pain, even in the presence of significant deformities, are unlikely to benefit from surgery. Those patients with degenerative disease at the wrist, or DRUJ, should be considered for salvage surgery rather than corrective osteotomy [38].

Approach and technique

Techniques have evolved over the past 20 years, from the use of a dorsal approach and fixation with non-locking plates, or Kirschner wires (K-wires), towards a volar approach and fixation with a fixed angled anatomical locking plate in the majority of situations [7,42,43]. The choice of technique is determined by the configuration and magnitude of the deformity and the presence of concurrent pathology. An opening wedge osteotomy is preferred to a closing wedge technique to avoid exacerbations of radial shortening.

The approach is made through either a volar incision through the bed of the flexor carpi radialis or a dorsal incision and dissection between the third and fourth extensor compartments. Radial osteotomies are made with an oscillating saw, and cooled with normal saline to minimise heat necrosis or osteotomies. A cut is usually made at the apex of the deformity and site of the original fracture or as a line parallel with the distal articular surface. To aid correction, the release of the brachioradialis may be performed. The radial shaft can be cleared of excessive callus dorsally; this can be achieved through a volar approach by delivering the pronated radius into the volar wound.

While no single fixation device has been shown to have superior results, most authors agree that fixed-angle devices are superior in their ability to maintain correction with sufficient strength and stability at the osteotomy site to enable rehabilitation during bone healing [25,44-45]. External fixation is not widely used in the management of closed distal radius fractures [3]. McQueen et al. reported a complication rate of 57% with this technique for corrective osteotomy, including a 9% rate of extensor pollicis longus (EPL) rupture [6].

Deformity correction through a volar approach has gained popularity in recent years because it reduces the risk of extensor tendon rupture and metal work irritation. Schweizer et al. also reported favourable wrist flexion with a volar approach, hypothesising there may be less tendon irritation and soft tissue scarring [46]. The authors report, however, that patients with >5mm of positive ulnar variance are difficult to fully correct through a volar approach [14], with 19%-60% of patients requiring subsequent ulnar shortening procedures [7,16,38,47].

A dorsal approach allows greater access to the void created by osteotomy in dorsal malunion. For surgeons using a structural graft, these can be more easily shaped and positioned via a dorsal approach. Perhaps due to greater dorsal soft tissue release, larger corrections of ulnar variance are possible. It may also be indicated in cases where dorsal pathology, such as scapholunate ligament injury, is to be treated simultaneously [46].

Newer dorsal plating systems are aimed at reducing the risk of extensor rupture; however, tendon irritation and damage remain the biggest problems. The rupture rates of EPL are reported to be as high as 10% [16, 48], and the need for metalwork removal is 63% versus 36% for volar fixation [46,49]. An alternative to achieving lengthening greater than 1cm via a dorsal approach is a simultaneous ulnar shortening osteotomy [23,34-35].

Ulnar corner procedures

Patients with gross instability at the DRUJ should be considered for stabilisation. Methods for this include fixation of ulnar styloid non-unions, TFCC repair, and Adams tenodesis [19]. In cases with advanced degenerative changes, salvage procedures may be considered (Table 1).

**Table 1:** Surgical solutions to DRUJ pathology DRUJ: distal radioulnar joint; TFCC: triangular fibrocartilage complex; OA: osteoarthritis

Ulnar corner pathology	Surgical reconstructive options
Instability	Arthroscopic or open TFCC repair/ulnar styloid fixation/Adams tenodesis
DRUJ OA	Ulnar head replacement/Sauvé-Kapandji/modified Bowers procedure/Darrachs (lower functional demand)

Indications for void filler or bone graft

Strategies to manage the void left by corrective osteotomy can be classified as either (a) no graft, (b) autologous bone graft, or (c) synthetic bone substitute. While a review article concluded that no bone graft is needed, this perhaps only applies to smaller corrections in which there remains some contact between bone fragments following osteotomy [50]. The magnitude of the correction is likely to influence the requirement for a bone graft. Scheer et al. had to stop their randomised trial early due to a 50% non-union rate in six patients [50] who had trapezoidal-shaped defects following correction.

Insufficient evidence exists to support the use of autologous grafts over synthetic bone substitutes. The iliac crest is the preferred autologous donor site from which to harvest the large volumes (3-5 cm3) of bone required. Some authors report a lower time to union with the iliac crest than synthetic alternatives, but there is no evidence to support the use of structural over non-structural grafts. Donor site morbidity is high, with complications including pain, seroma, infection, nerve injury, ureteric injury, and haematoma, with an additional operative time of around 2020 minutes [51].

Two commonly used compounds of the synthetic bone substitute are calcium sulphate and calcium phosphate cement. Calcium sulphate alone is felt to be unsuitable for use due to the high speed of resorption. Whereas the use of the structurally superior calcium phosphate cement, hydroxyapatite (HA), takes years to resorb and solidifies in a manner that may inhibit bony ingrowth or may cause stress shielding due to differences in Young’s modulus [29]. Tarrallo et al. reported that the majority of calcium phosphate cement was still present two years postoperatively in their patients and felt that surrounding healing bone “at best incorporates and bridges the void filler” [14]. One study reportedly used a product containing a mixture of 60% calcium sulphate and 40% calcium phosphate, achieving a union rate of 96% [5].

Outcome

Despite the technical challenges associated with the procedure, patient satisfaction following correction is high [16,28]. The majority of authors report significant improvements in pain, with gains of 3.1-4 points on a 10-point visual analogue pain scale (VAS) and clinically significant improvements in Quick DASH, Mayo, and SF-12 scores [16,25,28]. The mean time to union varies between 8 and 23 weeks.

In some series, authors reported dramatic improvements in the range of rotational motion (15-55 degrees), although others found no improvements [5,29]. Studies reporting grip strength measurements found improvements of up to 26 Newtons [25]. An increase in strength from 61% pre-operatively to 85% post-operatively, when compared to the contralateral uninjured side, is reported [28]. Winge et al. found similar gains but reported that rehabilitation to this level took six months [29].

The overall complication rate for radial osteotomy varies from 27% to 57% depending on the technique and the definition of complication [5,6,28]. Non-union is reported to occur in 0%-10.5% of cases, although rates vary across the literature even in series using bone graft or bone substitute. Higher rates of non-union occur in smokers [44]. Rates of complex regional pain syndrome (CRPS) range from 2.5% to 4.4% [44].

The most commonly encountered complication from dorsal fixation was extensor tendon irritation or rupture, necessitating metalwork removal in up to 69% of patients [52].

Complications occurring in volar-sided surgery are related to metalwork failure (10%), loss of position, and the need for subsequent ulnar shortening in up to 60% of cases [28,47]. Also reported are high rates of median neuropathy following correction [44,51]. This perhaps arises from techniques that use locking T-plates to create a radially deviated epiphysis prior to making the osteotomy, causing excessive pressure on the nerve.

## Conclusions

A fundamental understanding of wrist biomechanics is necessary to understand patterns of deformity and their consequences. Distal radial osteotomy should be reserved for symptomatic patients with angular radial deformity, in addition to shortening. Patients with degenerative joint disease may be best managed with salvage techniques. In general, the quality of the evidence is poor, but it supports the use of a volar approach without bone graft for modest corrections and the use of bone graft or synthetic substitute when a trapezoidal-shaped void is left following correction. Despite a high complication rate, patient satisfaction rates as measured by objective measures and functional outcome scores are often much improved.
